# Developing a Framework for Safe AI Model Development on Sensitive Healthcare Data

**DOI:** 10.23889/ijpds.v9i5.2521

**Published:** 2024-09-18

**Authors:** Lewis Hotchkiss, Emma Squires, Timothy Rittman, John Gallacher, Simon Thompson

**Affiliations:** 1 Swansea University; 2 University of Cambridge; 3 University of Oxford

The Dementias Platform UK (DPUK) Data Portal holds over 60 cohort datasets from over 3 million participants with a range of multi-modal data including neuroimaging and genomics. This has meant we have seen an increasing interest in the development of AI models with the potential for clinical implementation. However, this presents a unique challenge to disclosure control when it comes to assessing these AI models for release due to the risk of attacks such as membership inference, model inversion or even just vulnerabilities in the models like overfitting. This is why we hosted a series of workshops bringing together members of the public, expert researchers, and data owners across the UK to build a framework for allowing the safe development and release of AI models trained on sensitive healthcare data. From this, we have put together recommendations and guidelines for the use of privacy-preserving techniques in AI models to protect patient privacy, and to allow the safe deployment of these models outside of trusted research environments. We also identified the unique challenges to privacy and implementation of privacy-preserving techniques in AI models regarding the use of complex data such as neuroimaging and genomics. This framework has created a path forward for supporting safe AI model development which takes into consideration rapidly evolving ethical, legal and privacy considerations.

